# Binary Phase Behavior of 1,3-Distearoyl-2-oleoyl-*sn*-glycerol (SOS) and Trilaurin (LLL)

**DOI:** 10.3390/molecules25225313

**Published:** 2020-11-14

**Authors:** Shinichi Yoshikawa, Shimpei Watanabe, Yoshinori Yamamoto, Fumitoshi Kaneko

**Affiliations:** 1 Research Institute for Creating the Future, Fuji Oil Holdings Inc., Izumisano 598-8540, Japan; yoshikawa.shinichi@so.fujioil.co.jp (S.Y.); watanabe.shimpei@so.fujioil.co.jp (S.W.); 2 Graduate School of Science, Osaka University, Toyonaka 560-0043, Japan; yamamotoy16@chem.sci.osaka-u.ac.jp

**Keywords:** 1,3-distearoyl-2-oleoyl-*sn*-glycerol (SOS), trilaurin (LLL), eutectic, solid solution, phase separation, polymorph, differential scanning calorimetry, synchrotron radiation X-ray diffractometry

## Abstract

This paper reports the precise analysis of the eutectic mixing behavior of 1,3-distearoyl-2-oleoyl-*sn*-glycerol (SOS) and trilaurin (LLL), as a typical model case of the mixture of cocoa butter (CB) and cocoa butter substitute (CBS). SOS was mixed with LLL at several mass fractions of LLL (*w*_LLL_); the mixtures obtained were analyzed for polymorphic phase behavior using differential scanning calorimetry (DSC) and synchrotron radiation X-ray diffractometry (SR-XRD). In melt crystallization with constant-rate cooling, SOS and LLL formed eutectics in their metastable polymorphs, allowing the occurrence of a compatible solid solution at *w*_LLL_ ≥ 0.925. With subsequent heating, the resultant crystals transformed toward more stable polymorphs, then melted in a eutectic manner. For mixtures aged at 25 °C after melt crystallization, eutectics were found in the extended *w*_LLL_ region, even at *w*_LLL_ = 0.975. These results indicate that phase separation between SOS and LLL progressed in their solid solution under stabilization. The crystal growth of the separated SOS fraction may cause fat-bloom formation in compound chocolate containing CB and CBS. To solve this problem, the development of retardation techniques against phase separation is expected.

## 1. Introduction

Cocoa butter (CB), a material fat for chocolate products, is obtained from the beans of the cacao plant (*Theobroma cacao* L.) by press, expeller, or solvent extraction [1,2,3]. The predominant constituents are about 95% to 97% triacylglycerols (TAGs) enriched with stearic acid (S, C18:0), oleic acid (O, C18:1), and palmitic acid (P, C16:0) as the major fatty-acid moieties. Unlike most naturally occurring fats, the symmetrical 1,3-disaturated-2-oleoyl-*sn*-glycerols of 1(3)-palmitoyl-2-oleoyl-3(1)-stearoyl-*sn*-glycerol (POS), 1,3-distearoyl-2-oleoyl-*sn*-glycerol (SOS), and 1,3-dipalmitoyl-2-oleoyl-*sn*-glycerol (POP) comprise at least 70% of the total TAGs in CB [3,4,5,6,7]. Due to this unique TAG composition, CB is solid at temperatures below ~25 °C and liquid at the body temperature of ~37 °C, resulting in sharp melting in the mouth [8,9]. Moreover, CB presents six crystal polymorphs defined by sub-cell and chain-length structure: form I (sub α-2), form II (α-2), form III (β′_2_-2), form IV (β′_1_-2), form V (β_2_-3), and form VI (β_1_-3) in ascending order of thermal stability [4,10,11]. Above all, fine crystals of CB in form V provide chocolate products with such desirable qualities as smooth mouthfeel, heat resistance, maximum contraction for easy demolding, consistency for good snap, sheeny appearance, and fat-bloom resistance for prolonged shelf life [12,13,14]. Although categorized as the fat of β-polymorphic tendency [15,16], CB requires a tempering (pre-crystallization) process for controlled crystallization in form V [10,13,14,17].

The unstable supply, high price, and uncertain quality of CB have resulted in the production of alternatives derived from other vegetable oils [18,19,20,21]. For example, cocoa butter substitute (CBS) has been developed principally by the fractionation and/or hydrogenation of lauric fats such as palm kernel oil (PKO) and coconut oil (CO) [21,22,23,24]. Therefore, the typical CBS contains a significant amount of lauric acid (L, C12:0) and myristic acid (M, C14:0), which constitute the major TAGs of trilaurin (LLL), 1,2(2,3)-dilauroyl-3(1)-myristoyl-*sn*-glycerol (LLM), and 1(3)-lauroyl-2,3(1,2)-dimyristoyl-*sn*-glycerol (LMM) [17,18,22]. The coexistence of these high-melting TAGs enables CBS to crystallize directly from the melt in the stable β′-2 polymorph, so that tempering is not required for compound chocolate dominantly containing CBS in the fat phase [20,22,23]. However, the most stable β-2 polymorph was separately reported for LLL, LLM, and LMM, indicating the potential of CBS to form crystals of the β polymorph [25,26]. Indeed, lauric fats gradually transform from β′ to β in long-term storage [27,28,29].

Despite their similar physical properties, CB and CBS incompatibly form eutectics in their mixtures due to the significant difference in TAG composition [9,21,22,23,24]. In contrast with the single use of CB or CBS, such eutectic interaction induces the depression in the melting point, solid fat content (SFC), and hardness [7,11,30,31,32,33], which may cause processing problems and the quality degradation of compound chocolate [24]. A more serious effect is that fat bloom appears on the chocolate surface as a result of the development of a new solid phase separating from the base chocolate [33,34,35].

Phase diagrams are useful to clarify the interactions between two components of binary mixtures [36,37]. The eutectic interaction between CB and CBS is reflected in the characteristic pattern of the binary phase diagram that was previously constructed with the data of differential thermal analysis (DTA) and X-ray diffractometry (XRD) [38]. In this diagram, phase-transition temperatures determined by DTA were plotted as a function of the CBS fraction; moreover, crystal polymorphs with sub-cell and chain-length structure were identified using XRD to differentiate the solid phases. This diagram indicates that CB and CBS formed not only eutectics but also solid solutions for their partial compatibility. Specifically, CB mixed with less than ~5% CBS formed a solid solution in the β-3 polymorph after being subjected to aging. Likewise, the CBS mixed with less than ~5% CB formed a solid solution in the β′-2 polymorph. In contrast, the mixtures with no less than ~5% CB and CBS formed eutectics of the β-3 and β′-2 polymorphs. The boundaries between the solid solutions and the eutectics represent the solid-solubility limit of CB and CBS in the binary system [23]. Considering the ~5% solid-solubility limit, manufacturers of CBS-based compound chocolate control the CB level of the fat phase below 5% to prevent the occurrence of eutectic effects [22,24]. However, a higher solid-solubility limit is implied for less stable mixtures of CB with CBS, though the boundaries between the solid solutions and the eutectics remain unclear due to experimental difficulties [38]. Thus far, little has been determined regarding the change of the solid-solubility limit and its influence on eutectic effects.

At present, the mechanisms of eutectic formation between CB and CBS have not yet been determined, possibly because few fundamental studies on the mixing phase behavior of the component TAGs have been conducted. This study aimed to clarify the mixing phase behavior of SOS and LLL as a typical model case of the mixture of CB and CBS. For this purpose, mixtures of SOS and LLL (SOS/LLL) were precisely analyzed for crystallization kinetics and polymorphic phase behavior. Table 1 summarizes the crystal polymorphs of SOS and LLL with the corresponding melting points and structural information [39,40,41]. As combinations of a 2-oleoyl-1,3-disaturated mixed-acid TAG with a trisaturated monoacid TAG, SOS/tristearin (SSS), SOS/tripalmitin (PPP), and POP/PPP were previously examined for binary phase behavior; and all indicated the formation of incompatible monotectics [42,43]. The present study was the first to analyze the binary phase behavior of SOS/LLL.

Samples for all experiments in this study were prepared by mixing SOS with LLL at several mass fractions of LLL (*w*_LLL_). For example, the mixture at *w*_LLL_ = 0.667 contains SOS and LLL at a mass ratio of SOS/LLL = 0.333/0.667. Differential scanning calorimetry (DSC) and synchrotron radiation XRD (SR-XRD) were employed to analyze the polymorphic crystallization kinetics of the mixtures during constant-rate cooling (2 °C/min) and subsequent heating (5 °C/min). Before cooling, the mixtures were pretreated at 80 °C for 10 min to ensure the molten state, eliminating any crystal memory. The following aging process was inserted as necessary between the cooling and heating: cooling at 15 °C for 30 min, heating to 25 °C at a rate of 5 °C/min, and then maintaining the temperature for two weeks.

## 2. Results

### 2.1. Crystallization Kinetics Analysis Using DSC

**Figure 1:**
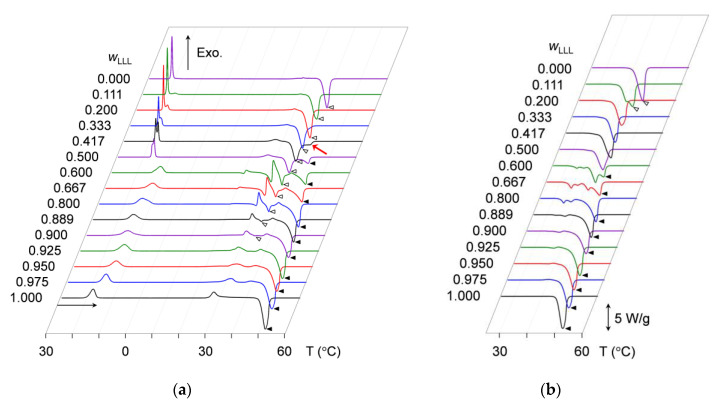
Differential scanning calorimetry (DSC) thermograms of mixtures of SOS with LLL (SOS/LLL) at several mass fractions of LLL (*w*_LLL_). Open triangles denote endothermic peaks for SOS at the highest peak-top temperature, and closed triangles denote those for LLL. (**a**) Thermograms taken during constant-rate cooling (2 °C/min) and subsequent heating (5 °C/min); (**b**) thermograms taken during constant-rate heating (5 °C/min) after aging at 25 °C for two weeks.

DSC measurements were conducted to examine the crystallization kinetics of the mixtures in terms of heat flow changing with varying temperature during cooling and heating. DSC thermograms were acquired by continuously converting the temperature difference between the sample and empty pans to the heat flow. In the thermograms, exothermic peaks due to the heat of crystallization and endothermic peaks due to the heat of fusion were analyzed to determine peak-top temperatures. For identical samples, the measurement was repeated at least three times. Figure 1 presents representative thermograms of all the mixtures. The peak-top temperatures in the multiple measurements were averaged to define phase-transition temperatures of the mixtures. Average values of the peak-top temperatures are summarized in Table S1.

#### 2.1.1. Cooling Melt-Crystallization Kinetics

Figure 1a depicts the thermograms taken during the cooling-and-heating without applying the aging process. In the left part of Figure 1a, the cooling thermograms at 30 to 0 °C indicate the occurrence of exothermic peaks due to the melt crystallization of SOS and LLL. However, the peak pattern clearly differs between the mixtures at *w*_LLL_ = 0.000 to 0.500 and those at *w*_LLL_ = 0.600 to 1.000.

For SOS-dominant mixtures at *w*_LLL_ = 0.000 to 0.500, the cooling thermograms display one or two sharp exothermic peaks. By associating peak intensity with *w*_LLL_ values, the peak at a higher temperature is attributed to the crystallization of SOS, and that at a lower temperature is attributed to the crystallization of LLL. As the *w*_LLL_ values increase, the two peaks reduce their peak-top temperatures from 22.6 °C (*w*_LLL_ = 0.000) to 17.5 °C (*w*_LLL_ = 0.500) for SOS and from 17.9 °C (*w*_LLL_ = 0.111) to 17.0 °C (*w*_LLL_ = 0.500) for LLL. Separation between the two peaks indicates the formation of incompatible eutectics between SOS and LLL.

In contrast, the LLL-dominant mixtures at *w*_LLL_ = 0.600 to 1.000 exhibit one broad peak alone in their cooling thermograms. With increasing *w*_LLL_ value, this peak increases its peak-top temperature from 11.6 °C (*w*_LLL_ = 0.600) to 18.0 °C (*w*_LLL_ = 1.000). The similar peak shape between the mixtures at *w*_LLL_ = 0.600 to 0.975 and pure LLL at *w*_LLL_ = 1.000 implies the possibility that LLL crystallized compatibly incorporating the minor fraction of SOS to form a solid solution.

#### 2.1.2. Crystallization-Melting Kinetics during Heating without Aging

Immediately after the completion of cooling to 0 °C, the mixtures were reheated with constant-rate heating. As indicated in the right part of Figure 1a, the heating thermograms at 0 to 60 °C present complex peak patterns of the mixed exothermic and endothermic peaks, meaning the sequential occurrence of crystallization and melting due to phase transitions in the solidified mixtures. For example, the mixture at *w*_LLL_ = 0.667 exhibits as many as seven peaks: a faint endothermic peak at 19.3 °C, a small exothermic peak at 20.6 °C, an endothermic peak at 29.7 °C, a large exothermic peak at 31.0 °C, an endothermic peak at 33.9 °C, a small exothermic peak at 37.6 °C, and a large endothermic peak at 44.5 °C.

In contrast, only two peaks are observed in the heating thermograms of pure SOS at *w*_LLL_ = 0.000 and pure LLL at *w*_LLL_ = 1.000; one is a minor exothermic peak at 27.1 to 27.9 °C; the other is a main endothermic peak at the higher temperature. This endothermic peak is ascribed to the melting of SOS or LLL crystals. Corresponding endothermic peaks are also observed for the selected mixtures, as indicated by open and closed triangles in Figure 1a. The endothermic peak for the melting of SOS crystals, found in mixtures at *w*_LLL_ = 0.000 to 0.900, exhibits a peak-top temperature ranging from 36.2 °C (*w*_LLL_ = 0.000) to 33.4 °C (*w*_LLL_ = 0.900). In contrast, the endothermic peak for the melting of LLL crystals, found in mixtures at *w*_LLL_ = 0.500 to 1.000, exhibits a peak-top temperature ranging from 41.4 °C (*w*_LLL_ = 0.500) to 45.8 °C (*w*_LLL_ = 1.000).

#### 2.1.3. Crystallization-Melting Kinetics during Heating after Aging

Figure 1b depicts the heating thermograms of mixtures that underwent aging at 25 °C for two weeks after cooling melt crystallization. Identical thermograms were obtained when the aging period was extended to one month (data not shown). In the same manner as the non-aged mixtures, aged mixtures at *w*_LLL_ = 0.600 and 0.667 exhibit more peaks than the other aged mixtures. For example, the mixture at *w*_LLL_ = 0.667 exhibits four endothermic peaks at 33.3, 35.8, 39.5, and 43.5 °C; the former three peak-top temperatures are higher than those of the corresponding endothermic peaks observed for the non-aged mixture at *w*_LLL_ = 0.667. In contrast with the non-aged mixtures, no marked exothermic peaks are found for the aged mixtures.

Pure SOS at *w*_LLL_ = 0.000 exhibits a single endothermic peak at 41.8 °C, and pure LLL at *w*_LLL_ = 1.000 exhibits a single endothermic peak 46.1 °C. Corresponding peaks due to the melting of SOS and LLL crystals are also observed for the selected mixtures, as indicated by open and closed triangles in Figure 1b. The endothermic peak for the melting of SOS crystals, found in mixtures at *w*_LLL_ = 0.000 and 0.111, exhibits a peak-top temperature ranging from 41.8 °C (*w*_LLL_ = 0.000) to 41.1 °C (*w*_LLL_ = 1.000). In contrast, the endothermic peak for the melting of LLL crystals, found in mixtures at *w*_LLL_ = 0.600 to 1.000, exhibits a peak-top temperature ranging from 42.7 °C (*w*_LLL_ = 0.600) to 46.1 °C (*w*_LLL_ = 1.000). The comparison of the heating thermograms between non-aged (right part of Figure 1a) and aged (Figure 1b) mixtures indicates that these endothermic peaks increase peak-top temperatures with aging.

These results indicate that aging caused the polymorphic stabilization of SOS and LLL crystals in the mixtures employed, hence simple melting with little recrystallization in subsequent heating. The plural endothermic peaks in the heating thermograms of the aged mixtures at *w*_LLL_ = 0.600 to 0.800 imply the unique situation that metastable crystals can survive in long-term storage.

### 2.2. Polymorphic Phase-Transition Analysis Using SR-XRD

For the further analysis of crystallization kinetics from the viewpoint of crystal structure, we performed SR-XRD measurements of the mixtures under the same thermal conditions as the DSC measurements. Because of the high-flux X-ray source and the high-sensitivity detector, SR-XRD enables the time-resolved monitoring of the polymorphic phase transition of TAG crystals [41,44]. In order to obtain a wide range of structural information, we carried out simultaneous measurements of a small-angle X-ray scattering (SAXS) and a wide-angle X-ray scattering (WAXS). TAGs form multi-layered lamellar structure in crystalline state, typically represented by a double or triple chain-length structure; the chain-length structure can be identified from long spacing that the SAXS profile provides. The short spacing of a repetition unit between the adjacent hydrocarbon chains of TAG molecules is characterized by the WAXS profile, which defines the sub-cell structure unique to each crystal polymorph. Spacing *d* is determined as a reciprocal of the magnitude of the scattering vector |*s*| (*d* = 1/|*s*| = *λ*/(2sin*θ*)), where *θ* is the scattering angle and *λ* is the wavelength of X-rays applied [45]. The TAG species and polymorphs of the crystals occurring in the mixtures were identified by comparing the experimental *d* values with the corresponding values in Table 1.

#### 2.2.1. Cooling-and-Heating Phase Transition (*w*_LLL_ = 0.950)

**Figure 2:**
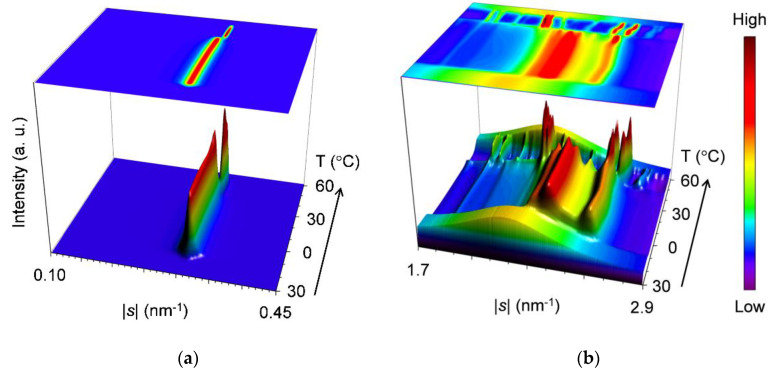
Topographic plots of the synchrotron X-ray diffractometry (SR-XRD) data for SOS/LLL (*w*_LLL_ = 0.950), taken during the cooling at a rate of 2 °C/min and subsequent heating at a rate of 5 °C/min: (**a**) small-angle X-ray scattering (SAXS); and (**b**) wide-angle X-ray scattering (WAXS).

Figure 2 shows results of the SR-XRD measurements performed on the mixture at *w*_LLL_ = 0.950 during the cooling-and-heating. The data are displayed three-dimensionally in topographical plots where the time-dependent variation of the SAXS (Figure 2a) and WAXS (Figure 2b) profiles are presented from front to back; moreover, the profiles are projected on the overhead surfaces.

**Figure 3:**
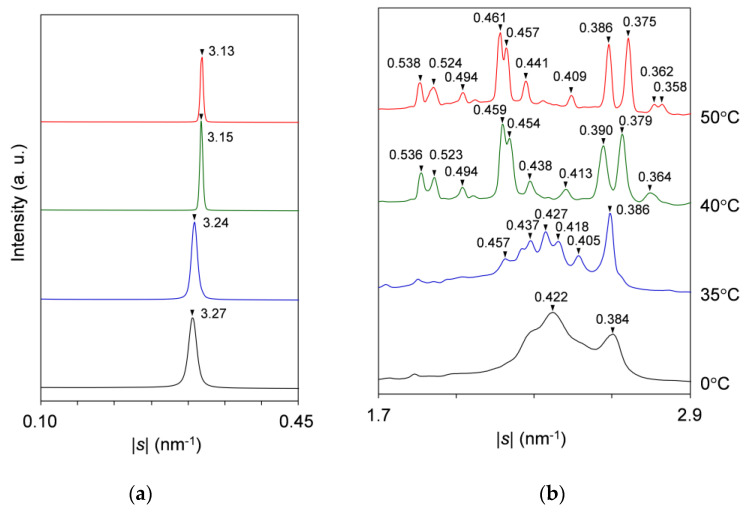
Representative SR-XRD profiles of SOS/LLL (*w*_LLL_ = 0.950) in the heating process of Figure 2: (**a**) SAXS; and (**b**) WAXS. Unit: nm.

For the SAXS profiles (Figure 2a), a single peak at |*s*| = 0.307 nm^−1^ (*d* = 3.25 nm) occurs first at ~21 °C in the cooling process, attributable to the crystallization of LLL in the β′-2 polymorph. As the temperature decreases, this peak sharpens, increasing its diffraction intensity until ~14 °C and then plateaus, preserving intensity and position. In subsequent heating, this peak grows again from ~21 °C and rapidly declines from ~34 °C before disappearing at ~39 °C. Meanwhile, the peak shifts its position slightly from |*s*| = 0.306 nm^−1^ (*d* = 3.27 nm) at 0 °C to |*s*| = 0.309 nm^−1^ (*d* = 3.24 nm) at 35 °C. Shortly afterward, a new peak at |*s*| = 0.318 nm^−1^ (*d* = 3.15 nm) appears from ~35 °C, ascribable to the occurrence of LLL β-2 crystals. With further heating, this peak develops until ~43 °C before disappearing at ~55 °C, exhibiting a slight positional shift to |*s*| = 0.319 nm^−1^ (*d* = 3.13 nm) at 50 °C. The extracted SAXS profiles at 0, 35, 40, and 50 °C in the heating process are presented in Figure 3a. It should be noted that the SAXS profiles offer no evidence for the occurrence of SOS crystals in the mixture.

Corresponding to the varying SAXS profiles, the WAXS profiles (Figure 2b) present four major peak patterns, which are summarized in Figure 3b. As typically observed at 0 °C, the first peak pattern has bimodal peaks at |*s*| = 2.37 nm^−1^ (*d* = 0.422 nm) and 2.61 nm^−1^ (*d* = 0.384 nm) with positional modification depending on the temperature. These peaks, characteristic of the β′ sub-cell structure, occur first at ~21 °C during cooling and start to separate at ~27 °C during subsequent heating.

The second peak pattern appears clearly at 33 to 35 °C during heating. As typically observed at 35 °C, this pattern is complicated with peaks at |*s*| = 2.19 nm^−1^ (*d* = 0.457 nm), 2.29 nm^−1^ (*d* = 0.437 nm), 2.34 nm^−1^ (*d* = 0.427 nm), 2.39 nm^−1^ (*d* = 0.418 nm), 2.47 nm^−1^ (*d* = 0.405 nm), and 2.59 nm^−1^ (*d* = 0.386 nm). The occurrence of these peaks was recently reported for LLL β′-2 crystals, which were obtained by melt crystallization with isothermal cooling at 28 °C [46]. Takeguchi et al. associated these peaks with the development of crystal perfection. Certainly, it can be assumed that these peaks cannot be observed at 0 °C due to the broadening caused by crystal imperfection and that they appear as clear-cut peaks at 35 °C due to the sharpening caused by the improvement in crystal perfection. However, the peak at |*s*| = 2.19 nm^−1^ (*d* = 0.457 nm) is specific to the β sub-cell structure and appears to be carried over to the corresponding peak at |*s*| = 2.18 nm^−1^ (*d* = 0.459 nm), which is observed with the intensified signal at 40 °C. Therefore, we conclusively ascribe the complex peak pattern at 35 °C to the coexistence of the LLL β′-2 crystals with improved perfection and the LLL β-2 crystals that converted from the imperfect LLL β′-2 crystals. The LLL β-2 crystals have potential to act as the subsequent crystallization of LLL in the β-2 polymorph.

The third peak pattern appears to be replacing the second one from 35 to 39 °C during heating. As typically observed at 40 °C, this pattern is characterized by major peaks at |*s*| = 2.18 nm^−1^ (*d* = 0.459 nm), 2.20 nm^−1^ (*d* = 0.454 nm), 2.57 nm^−1^ (*d* = 0.390 nm), and 2.64 nm^−1^ (*d* = 0.379 nm). These peaks, specific to the β sub-cell structure, and a split of the former two peaks were previously observed for the LLL β-2 crystals that occurred at the initial stage of crystallization under ultrasonication [47]. Since peak-pattern replacement progresses at a temperature above the melting point of LLL β′-2 crystals (Table 1), this event is reasonably attributed to the melt-mediated transformation of LLL crystals from β′-2 to β-2.

The fourth peak pattern is apparently derived from the third one as a result of peak shifts at 45 to 47 °C during heating. As typically observed at 50 °C, this pattern is characterized by major peaks at |*s*| = 2.17 nm^−1^ (*d* = 0.461 nm), 2.19 nm^−1^ (*d* = 0.457 nm), 2.59 nm^−1^ (*d* = 0.386 nm), and 2.66 nm^−1^ (*d* = 0.375 nm). Although the extent of the peak shifts is not as large, the overhead projection of the WAXS profiles (top surface in Figure 2b) clearly indicates that the former two peaks and the latter two peaks undergo peak shifts toward the lower and the higher |*s*|-value directions, respectively. Meanwhile, the latter two peaks weaken temporarily, and a new small peak occurs at |*s*| = 2.79 nm^−1^ (*d* = 0.358 nm). These changes indicate the rearrangement of the acyl chains in the sub-cell structure of the LLL β-2 crystals; the characteristic short spacings before and after the changes have recently been reported for pure LLL [48]. Therefore, we attribute these changes to the polymorphic transformation of LLL crystals from the less stable β-2 (β_2_-2) to the more stable β-2 (β_1_-2). Two subtypes of the β polymorph were previously reported for LLL β-2 crystals occurring in the mixture of LLL with cholesterol at the 4% molar fraction [49]. The same change in the WAXS profile was also reported for LLL β-2 crystals occurring under ultrasonication; however, Lee et al. contended that this change was due to the increase in crystal perfection caused by facets of late-occurring crystals [47]. To put an end to the controversy as to whether the LLL β-2 has a subtype or not, the precise structure determination of the less stable LLL β-2 crystals is needed. Finally, all the WAXS peaks disappear completely at ~55 °C.

Combining the SAXS and WAXS results, we summarized the polymorphic phase transitions of the mixture at *w*_LLL_ = 0.950 as follows. (1) During cooling, LLL starts to crystallize in the β′-2 polymorph at ~21 °C and grows until ~14 °C. (2) During subsequent heating, LLL β_2_-2 crystals occur at the partial expense of the LLL β′-2 crystals from ~21 °C, improving the crystal perfection of the residual LLL β′-2 crystals. (3) The remaining LLL β′-2 crystals melt from ~34 to ~39 °C and recrystallize as LLL β_2_-2 crystals from ~35 to ~43 °C. (4) The LLL β_2_-2 crystals melt from ~43 °C, partly converting to LLL β_1_-2 crystals from ~45 to ~47 °C. (5) The LLL β_1_-2 crystals melt completely at ~55 °C. These phase transitions are in good agreement with the thermal behavior in the DSC measurement (Figure 1a). The lack of diffraction peaks due to SOS crystals in the SAXS profiles suggests that the minor fraction SOS was incorporated in the LLL crystals as a solid solution.

#### 2.2.2. Cooling-and-Heating Phase Transition (*w*_LLL_ = 0.667)

**Figure 4:**
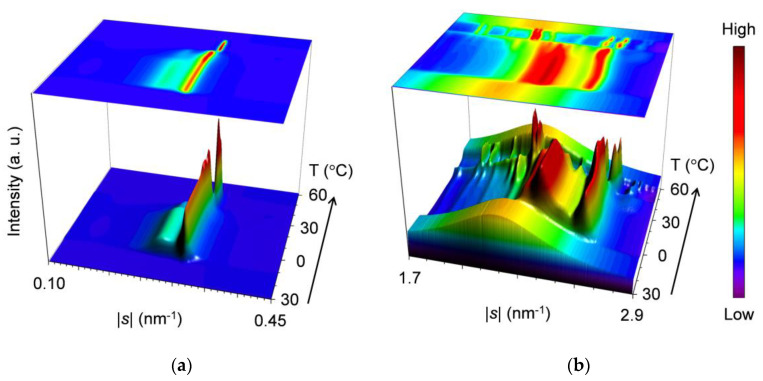
Topographic plots of the SR-XRD data for SOS/LLL (*w*_LLL_ = 0.667), taken during the cooling at a rate of 2 °C/min and subsequent heating at a rate of 5 °C/min: (**a**) SAXS; and (**b**) WAXS.

Figure 4 presents topographic plots of the SR-XRD data for the mixture at *w*_LLL_ = 0.667, taken during cooling and heating.

For the SAXS profiles in Figure 4a, a diffuse peak appears in two stages at the lower |*s*| position of a sharp peak at |*s*| = 0.304 nm^−1^ to 0.307 nm^−1^ (*d* = 3.29 nm to 3.25 nm). From the position at |*s*| = 0.266 nm^−1^ to 0.271 nm^−1^ (*d* = 3.76 nm to 3.69 nm), the diffuse peak indicates the occurrence of SOS crystals. Since the sharp peak behaves in the same manner as that observed for the mixture at *w*_LLL_ = 0.950, this peak is associated with LLL crystals.

During cooling, a single peak at |*s*| = 0.304 nm^−1^ (*d* = 3.29 nm) occurs first at ~18 °C, indicating the crystallization of LLL in the β′-2 polymorph. This peak sharpens, increasing its diffraction intensity from ~18 to ~10 °C, and then plateaus, preserving intensity and position. Shortly after the occurrence of the peak for the LLL β′-2 crystals, a bulge appearing on the lower |*s*| side of this peak at ~14 °C grows to a diffuse peak at |*s*| = 0.266 nm^−1^ (*d* = 3.76 nm) with its plateaued maximum intensity at ~10 °C. Considering the fact that the diffuse peak weakens from ~10 °C during subsequent heating, we attribute this peak to the transitional structure of SOS α_2_-(2 + 3) crystals reorganizing the acyl-chain packing from hexagonal to orthorhombic. The α_2_-(2 + 3) phase, having mixed double and triple chain-length structure, was defined as the less stable subtype of the α polymorph together with the more stable α_1_-2 [50].

**Figure 5:**
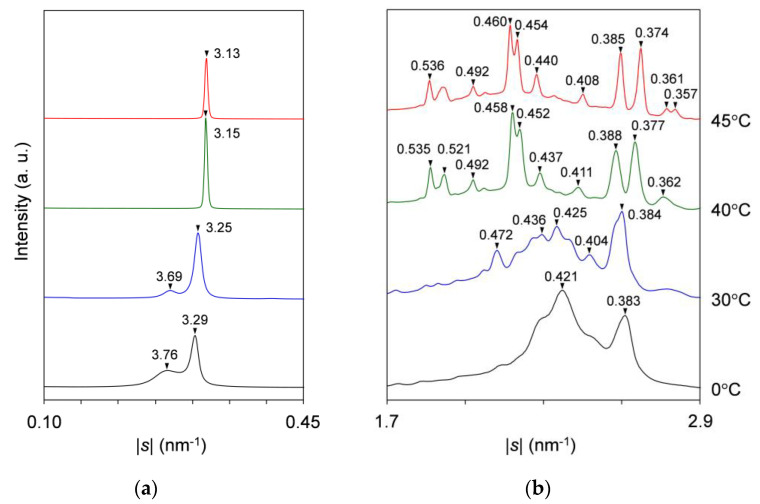
Representative SR-XRD profiles of SOS/LLL (*w*_LLL_ = 0.667) in the heating process of Figure 4: (**a**) SAXS; and (**b**) WAXS. Unit: nm.

During heating, the SAXS-profile change begins with the attenuation of the diffuse peak from ~10 to ~24 °C, resulting in a less-intensified diffuse peak at |*s*| = 0.271 nm^−1^ (*d* = 3.69 nm). The resultant peak, attributed to the 002 reflection of SOS γ-3 crystals, weakens again without a break and disappears at ~35 °C. While the previously occurring diffuse peak attenuates from ~10 to ~24 °C, the sharp peak for LLL β′-2 crystals grows again from ~18 °C and declines from ~24 °C before disappearing at ~35 °C; in the meantime, the peak shifts position slightly from |*s*| = 0.304 nm^−1^ (*d* = 3.29 nm) at 0 °C to |*s*| = 0.307 nm^−1^ (*d* = 3.25 nm) at 30 °C. Shortly afterward, a new peak at |*s*| = 0.318 nm^−1^ (*d* = 3.15 nm) occurs from ~34 °C, ascribable to the occurrence of LLL β-2 crystals. With further heating, this peak develops until ~38 °C before disappearing at ~50 °C, exhibiting a slight positional shift to |*s*| = 0.319 nm^−1^ (*d* = 3.13 nm) at 45 °C. The extracted SAXS profiles at 0, 30, 40, and 45 °C during heating are presented in Figure 5a.

For the WAXS profiles in Figure 4b, their variation approximates that observed for the mixture at *w*_LLL_ = 0.950 (Figure 2b). The decisive difference is that a peak at |*s*| = 2.12 nm^−1^ (*d* = 0.472 nm), attributable to SOS γ-3 crystals, contaminates from ~11 °C during cooling and intensifies from ~14 to ~30 °C before disappearing at ~35 °C during subsequent heating. No trace for the transitional SOS α_2_-(2 + 3) crystals is found behind the strong peaks at |*s*| = 2.37 nm^−1^ (*d* = 0.421 nm) and 2.61 nm^−1^ (*d* = 0.383 nm), as typically observed at 0 °C (Figure 5b). These strong peaks due to LLL β′-2 crystals occur at ~15 °C during cooling and start to separate from ~20 °C during subsequent heating, exhibiting positional modification dependent on temperature. Subsequently, complex peaks due to the mixed SOS γ-3 crystals, LLL β′-2 crystals with improved perfection, and LLL β_2_-2 crystals emerge until their dissolution at ~35 °C, as typically observed at 30 °C. Around this time, peaks for the LLL β_2_-2 crystals grow from ~34 to ~38 °C. With further heating, peak shifts due to the polymorphic transformation of LLL crystals from β_2_-2 to β_1_-2 occur from ~41 to ~44 °C. Finally, all the WAXS peaks disappear completely at ~50 °C.

Combining the SAXS and WAXS results, we summarize the polymorphic phase transitions of the mixture at *w*_LLL_ = 0.667 as follows. (1) During cooling, LLL starts to crystallize in the β′-2 polymorph at ~18 °C. (2) During the growth of LLL β′-2 crystals from ~18 to ~10 °C, SOS starts to crystallize in the transitional α_2_-(2 + 3) polymorph at ~14 °C and in the γ-3 polymorph at ~11 °C. (3) During subsequent heating, the SOS α_2_-(2 + 3) and γ-3 crystals melt successively at ~10 to ~35 °C, accompanied by the recrystallization of SOS γ-3 starting from ~14 °C. (4) LLL β_2_-2 crystals occur at the partial expense of the LLL β′-2 crystals from ~18 °C, improving the crystal perfection of the residual LLL β′-2 crystals. (5) The remaining LLL β′-2 crystals melt from ~24 to ~35 °C and recrystallize as LLL β_2_-2 crystals from ~34 to ~38 °C. (6) The LLL β_2_-2 crystals melt from ~38 °C, partly converting to LLL β_1_-2 crystals from ~41 to ~44 °C. (7) The LLL β_1_-2 crystals melt completely at ~50 °C. These phase transitions are in good agreement with the thermal behavior in the DSC measurement (Figure 1a). Co-occurrence of the peaks for SOS and LLL crystals means eutectic formation in the mixture at *w*_LLL_ = 0.667.

#### 2.2.3. Cooling-and-Heating Phase Transition (*w*_LLL_ = 0.417)

Figure S1 presents topographic plots of the SR-XRD data taken for the equimolar mixture at *w*_LLL_ = 0.417 during cooling-and-heating. Peaks for SOS γ-3 crystals appear prominently in the SAXS (Figure S1a) and WAXS (Figure S1b) profiles: specifically, a SAXS peak from |*s*| = 0.267 nm^−1^ to 0.274 nm^−1^ (*d* = 3.74 nm to 3.64 nm) and WAXS peaks at |*s*| = 2.11 nm^−1^ to 2.12 nm^−1^ (*d* = 0.474 nm to 0.472 nm), 2.56 nm^−1^ to 2.58 nm^−1^ (*d* = 0.391 nm to 0.388 nm), 2.75 nm^−1^ to 2.79 nm^−1^ (*d* = 0.363 nm to 0.358 nm). In addition, weak SAXS peaks at |*s*| = 0.129 nm^−1^ to 0.132 nm^−1^ (*d* = 7.77 nm to 7.57 nm) and 0.403 nm^−1^ to 0.413 nm^−1^ (*d* = 2.48 nm to 2.42 nm) are concurrently observed as 001 and 003 reflections from the SOS γ-3 crystals. These peaks occur prior to the peaks for LLL β′-2 crystals during cooling. Variation in SAXS-peak intensity implies the temporary formation of transitional SOS α_2_-(2 + 3) crystals and the following recrystallization as SOS γ-3 crystals. Aside from these observations, the overall evolvement of the SAXS and WAXS profiles is similar for the mixtures at *w*_LLL_ = 0.417 and 0.667. The representative SAXS and WAXS profiles at 0, 30, 35, and 42 °C during heating are depicted in Figure S2.

From the SAXS and WAXS results, we summarize the polymorphic phase transitions of the mixture at *w*_LLL_ = 0.417 as follows. (1) During cooling, SOS starts to crystallize in the γ-3 polymorph at ~20 °C. (2) Following the rapid growth of SOS γ-3 crystals from ~20 to ~19 °C, LLL β′-2 crystals grow from ~19 to ~16 °C, and transitional SOS α_2_-(2 + 3) crystals grow from ~18 to 16 °C. (3) The SOS α_2_-(2 + 3) crystals gradually dissolve the structure from ~9 to 0 °C. (4) During subsequent heating, the SOS γ-3 crystals grow again from ~3 to ~13 °C before melting from ~28 to ~41 °C. (5) The LLL β′-2 crystals melt from ~28 to ~34 °C and recrystallize as LLL β_2_-2 crystals from ~32 to ~38 °C; this process includes the occurrence of LLL β′-2 crystals with improved perfection. (6) The LLL β_2_-2 crystals melt from ~39 °C, partly converting to LLL β_1_-2 crystals from ~41 to ~43 °C. (7) The LLL β_1_-2 crystals melt completely at ~46 °C. These phase transitions are in good agreement with the thermal behavior in the DSC measurement (Figure 1a). Co-occurrence of the peaks for SOS and LLL crystals means eutectic formation in the mixture at *w*_LLL_ = 0.417.

#### 2.2.4. Heating Phase Transition after Aging (*w*_LLL_ = 0.667)

**Figure 6:**
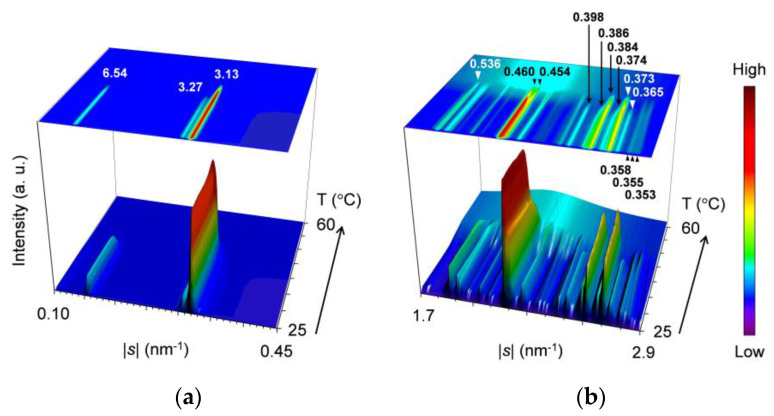
Topographic plots of the SR-XRD data for 25 °C-aged SOS/LLL (*w*_LLL_ = 0.667), taken during heating at a rate of 5 °C/min: (**a**) SAXS; and (**b**) WAXS. Unit: nm.

Aging after melt crystallization largely changes the polymorphic phase behavior of the mixtures during heating. Figure 6 presents the topographic plots of the SR-XRD data for the 25 °C-aged mixture at *w*_LLL_ = 0.667, taken during heating.

For the SAXS profiles in Figure 6a, three peaks at |*s*| = 0.153 nm^−1^ (*d* = 6.54 nm), 0.306 nm^−1^ (*d* = 3.27 nm), and 0.319 nm^−1^ (*d* = 3.13 nm) are observed at the initial stage of heating; the former two peaks are identified as 001 and 002 reflections of SOS β_2_-3 or β_1_-3 crystals, and the last intense peak is attributable to the 001 reflection of LLL β_2_-2 or β_1_-2 crystals, respectively. During heating, the SOS-specific peaks decrease their intensity from ~39 °C and disappear at ~43 °C. In contrast, the LLL-specific peak intensifies from ~35 to ~39 °C and then declines from ~41 °C before disappearing at ~48 °C.

For the WAXS profiles in Figure 6b, many peaks are observed at the initial stage of heating. These peaks are divided into two groups based on the disappearance temperatures of ~42 and ~48 °C. The group that disappears at the lower temperature consists mainly of peaks at |*s*| = 2.52 nm^−1^ (*d* = 0.398 nm), 2.59 nm^−1^ (*d* = 0.386 nm), 2.67 nm^−1^ (*d* = 0.374 nm), 2.74 nm^−1^ (*d* = 0.365 nm), and 2.82 nm^−1^ (*d* = 0.355 nm). The group that disappears at the higher temperature includes representative peaks at |*s*| = 1.86 nm^−1^ (*d* = 0.536 nm), 2.17 nm^−1^ (*d* = 0.460 nm), 2.20 nm^−1^ (*d* = 0.454 nm), 2.60 nm^−1^ (*d* = 0.384 nm), and 2.68 nm^−1^ (*d* = 0.373 nm). Because of the two-step decrement in peak intensity, a peak at |*s*| = 2.17 nm^−1^ (*d* = 0.460 nm) is assigned to both groups. Therefore, we presume from these *d* values that during heating, SOS β_2_-3 crystals and LLL β_2_-2 or β_1_-2 crystals melted in this order. Moreover, a peak at |*s*| = 2.84 nm^−1^ (*d* = 0.353 nm) gradually shifts its position to |*s*| = 2.82 nm^−1^ (*d* = 0.355 nm) until ~38 °C, intensifies from ~38 to ~40 °C, and then disappears at ~48 °C. This is consistent with the fact that the LLL-specific SAXS peak at |*s*| = 0.319 nm^−1^ (*d* = 3.13 nm) intensifies from ~35 to ~39 °C, indicating the occurrence of the polymorphic transformation of LLL crystals from β_2_-2 to β_1_-2.

To summarize the SR-XRD results for the 25 °C-aged mixture at *w*_LLL_ = 0.667, initially-coexisting SOS β_2_-3 and LLL β_2_-2 crystals undergo the polymorphic transformation of LLL crystals from β_2_-2 to β_1_-2 from ~35 to ~39 °C, the melting of SOS β_2_-3 crystals from ~39 to ~43 °C, and the melting of LLL β_1_-2 crystals from ~41 to ~48 °C.

The other aged mixtures were also analyzed for polymorphic phase transitions during heating. Although the SR-SRD data are not presented here, the representative results are summarized as follows. In the mixture at *w*_LLL_ = 0.950, LLL β_2_-2 crystals convert to LLL β_1_-2 crystals from ~37 to ~42 °C; SOS β_2_-3 crystals melt from ~37 to ~41 °C; and LLL β_1_-2 crystals melt from ~47 to ~52 °C. In the mixture at *w*_LLL_ = 0.417, LLL β_2_-2 and SOS β_2_-3 crystals melt simultaneously from ~40 to ~47 °C. In the mixture at *w*_LLL_ = 0.111, LLL β_2_-2 crystals melt from ~40 to ~43 °C, and SOS β_2_-3 crystals melt from ~40 to ~47 °C.

### 2.3. Solid-Solubility Limit of SOS in SOS/LLL

The SR-XRD data are also analyzed for the determination of the solid-solubility limit of SOS in SOS/LLL. In this study, the solid-solubility limit is defined as the maximum *w*_LLL_ value at which SAXS peaks or traces for SOS are confirmed. The less intense and denser WAXS peaks are not suitable for this analysis.

#### 2.3.1. Solid-Solubility Limit in Cooling Melt Crystallization

**Figure 7:**
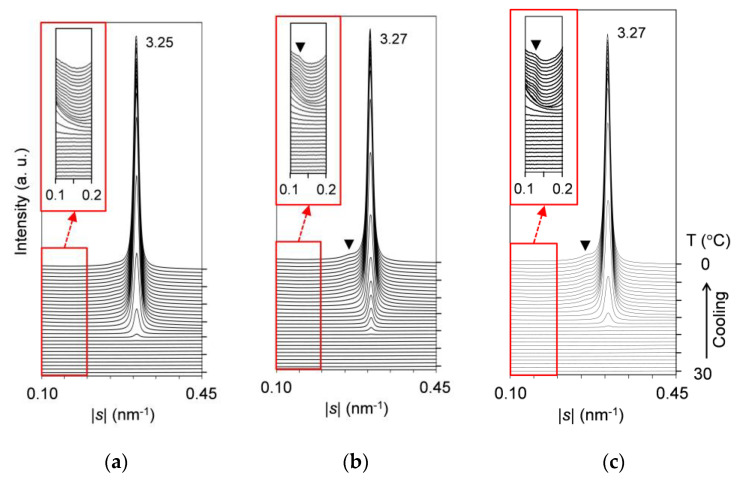
Time-resolved SAXS profiles of SOS/LLL, taken during cooling at a rate of 2 °C/min: (**a**) *w*_LLL_ = 0.925; (**b**) *w*_LLL_ = 0.900; and (**c**) *w*_LLL_ = 0.889. Insets depict the profiles with 50× magnified intensity. Unit: nm.

Figure 7 presents the comparative SAXS profiles of the mixtures at *w*_LLL_ = 0.925, 0.900, and 0.889, taken during melt crystallization with a constant-rate cooling (2 °C/min). Profiles are displayed every 1 °C, and the low-|*s*| parts of the profiles with magnified intensity are inserted as insets in this figure.

In Figure 7a, the mixture at *w*_LLL_ = 0.925 exhibits an intense peak at |*s*| = 0.307 nm^−1^ (*d* = 3.25 nm). This peak is due to LLL β′-2 crystals growing from the melt from ~21 to 0 °C. No other peaks or shoulders due to the crystallization of SOS are observed. This means that SOS is fully incorporated into the LLL β′-2 crystals as a solid solution.

In Figure 7b, the mixture at *w*_LLL_ = 0.900 also exhibits an intense peak at |*s*| = 0.306 nm^−1^ (*d* = 3.27 nm). Compared to the mixture at *w*_LLL_ = 0.925, this peak due to LLL β′-2 crystals is located at a slightly lower |*s*| position, although the first occurrence temperature is unchanged. In addition, two shoulders at |*s*| = ~0.127 nm^−1^ (*d* = ~7.87 nm) and ~0.261 nm^−1^ (*d* = ~3.83 nm) are observed from ~8 to 0 °C, as indicated by the closed triangle. These shoulders mean the occurrence of SOS crystals, because the corresponding peaks due to the 001 and 002 reflections from transitional SOS α_2_-(2 + 3) crystals were previously reported for pure SOS [50].

In Figure 7c, the mixture at *w*_LLL_ = 0.889 exhibits almost the same profiles as the mixture at *w*_LLL_ = 0.900. While the shoulders due to SOS crystals become more prominent, the peak due to LLL crystals starts at the lower temperature of ~19 °C.

We conclude from these results that in melt crystallization, the solid-solubility limit of SOS in SOS/LLL lies between *w*_LLL_ = 0.900 and 0.925. The continued data for subsequent heating at a rate of 5 °C/min indicate the unchanged solid-solubility limit (data not shown).

#### 2.3.2. Solid-Solubility Limit after Aging

**Figure 8:**
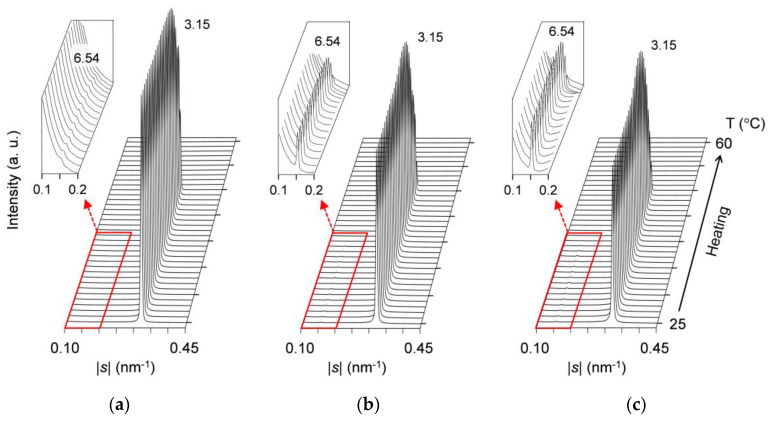
Time-resolved SAXS profiles of 25 °C-aged SOS/LLL, taken during heating at a rate of 5 °C/min: (**a**) *w*_LLL_ = 0.975; (**b**) *w*_LLL_ = 0.950; (**c**) *w*_LLL_ = 0.925. Insets depict the profiles with 150× magnified intensity. Unit: nm.

Figure 8 presents comparative SAXS profiles of the mixtures at *w*_LLL_ = 0.975, 0.950, and 0.925, taken during constant-rate heating (5 °C/min) after aging at 25 °C for two weeks. Profiles are displayed every 1 °C, and the low-|*s*| parts of the profiles with magnified intensity are inserted as insets in this figure. It should be noted that the SAXS profiles of the mixture at *w*_LLL_ = 0.925 (Figure 8c) are largely different from those taken during the cooling melt crystallization (Figure 7a).

These three mixtures all exhibit a weak peak at |*s*| = 0.153 nm^−1^ (*d* = 6.54 nm) and a strong peak at |*s*| = 0.318 nm^−1^ (*d* = 3.15 nm). The peak-intensity ratio of the weak peak to the strong peak rises with decreasing *w*_LLL_. As stated for the mixture at *w*_LLL_ = 0.950 in Section 2.2.4, the simultaneously obtained WAXS profiles indicate that LLL β_2_-2 crystals convert to LLL β_1_-2 crystals during heating. Therefore, at the initial stage of heating, the weak SAXS peak is identified as due to SOS β_2_-3 crystals, and the strong SAXS peak is identified as due to LLL β_2_-2 crystals. With elevating temperature, the weak peak disappears completely at ~41 °C, and the strong peak disappears completely at ~52 °C; these temperatures are constant among the three mixtures. Behind the strong peak, its shoulder at |*s*| = ~0.280 nm^−1^ (*d* = ~3.57 nm) is observed for only the mixture at *w*_LLL_ = 0.925.

These results confirm that after aging, SOS and LLL form eutectics in the mixture even at *w*_LLL_ = 0.975. That is, the solid-solubility limit of SOS in SOS/LLL lies between *w*_LLL_ = 0.975 and 1.000.

## 3. Discussion

### 3.1. Binary Phase Behavior of SOS/LLL

In this study, mixtures of SOS with LLL at the several mass fractions *w*_LLL_ were examined for crystallization kinetics, polymorphic phase transitions, and the solubility limit of SOS. Integrating all the results acquired, we construct diagrams for the binary phase behavior of SOS/LLL. In the diagrams, the phase-transition temperatures, defined as average values of the peak-top temperatures in the multiple DSC measurements (Table S1), are plotted as a function of *w*_LLL_.

#### 3.1.1. Cooling Melt-Crystallization Phase Behavior

**Figure 9:**
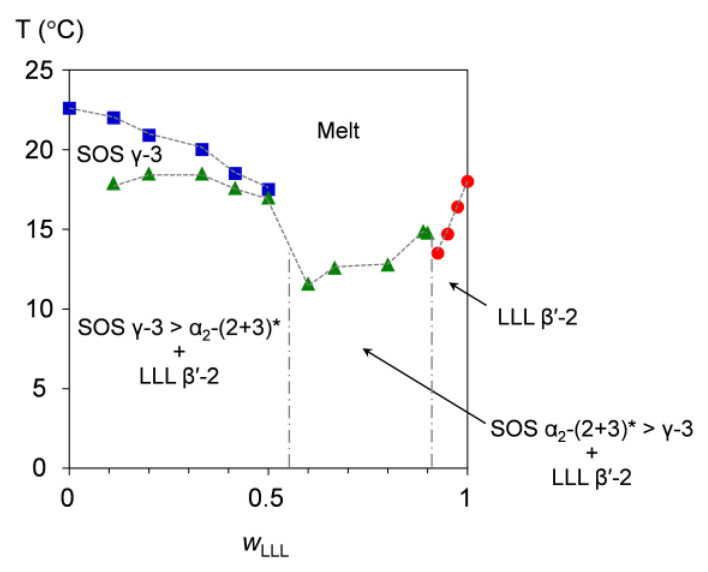
Melt-crystallization kinetic phase behavior of SOS/LLL as a function of *w*_LLL_, depicted for cooling at a rate of 2 °C/min. Symbols represent the phase-transition temperatures due to the exothermic effects of SOS (blue square), LLL (red circle), and SOS + LLL (green triangle). * Transitional structure of SOS α_2_-(2 + 3) is defined in reference [50].

Figure 9 presents the melt-crystallization kinetic phase behavior of SOS/LLL, depicted for a cooling at a rate of 2 °C/min. From the phase-transition behavior, this figure can be divided into three areas for the mixtures at *w*_LLL_ = 0.000 to 0.500, 0.600 to 0.900, and 0.925 to 1.000.

In the mixtures at *w*_LLL_ = 0.000 to 0.500, a large part of SOS crystallizes in the γ-3 polymorph at the initial stage of melt crystallization, as indicated by the phase-transition temperatures ranging from 22.6 °C (*w*_LLL_ = 0.000) to 17.5 °C (*w*_LLL_ = 0.500). Just below these temperatures, transitional SOS α_2_-(2 + 3) crystals and LLL β′-2 crystals occur at almost the same time, exhibiting nearly constant phase-transition temperatures from 17.0 to 18.5 °C.

In the mixtures at *w*_LLL_ = 0.600 to 0.900, three solid phases (transitional SOS α_2_-(2 + 3) crystals, SOS γ-3 crystals, and LLL β′-2 crystals) form from the melt at phase-transition temperatures ranging from 11.6 °C (*w*_LLL_ = 0.600) to 14.8 °C (*w*_LLL_ = 0.900). Among the three phases, the first-occurring phase is considered LLL β′-2 crystals, as observed for the mixture at *w*_LLL_ = 0.667 in the SR-XRD result (Figure 4). From this SR-XRD result, we extensively infer that SOS α_2_-(2 + 3) crystals probably surpass SOS γ-3 crystals in these mixtures.

In the mixtures at *w*_LLL_ = 0.925 to 1.000, only LLL β′-2 crystals occur from the melt at phase-transition temperatures ranging from 13.5 °C (*w*_LLL_ = 0.925) to 18.0 °C (*w*_LLL_ = 1.000). This indicates that the LLL β′-2 crystals grow incorporating the minor fraction of SOS as a solid solution.

#### 3.1.2. Heating Phase Behavior without Aging

**Figure 10:**
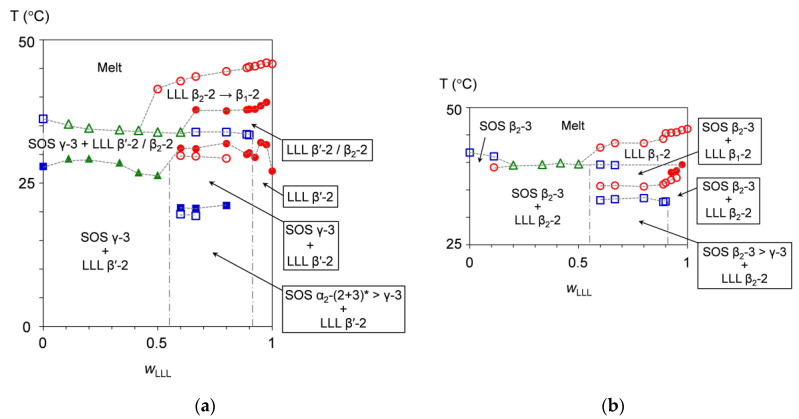
Phase behavior of SOS/LLL as a function of *w*_LLL_, depicted for heating at a rate of 5 °C/min: (**a**) without aging; and (**b**) after aging at 25 °C for two weeks. Closed symbols represent phase-transition temperatures due to exothermic effects, and open symbols represent those due to endothermic effects of SOS (blue square), LLL (red circle), and SOS + LLL (green triangle). * Transitional structure of SOS α_2_-(2 + 3) is defined in ref. [50].

Figure 10a (Figure 10b) presents the binary phase behavior of SOS/LLL, depicted for heating at a rate of 5 °C/min without (after) aging at 25 °C for two weeks. Regardless of whether or not the mixtures were exposed to aging before heating, each figure exhibits a depression from both sides, typically indicating eutectic formation between SOS and LLL.

In Figure 10a, the phase-transition behavior differs among the mixtures, seeming to inherit the melt-crystallization memory occurring in previous cooling. Just as the melt-crystallization kinetic phase behavior is divided into three areas (see Section 3.1.1), the heating phase behavior of the non-aged mixtures are correspondingly grouped into three areas at *w*_LLL_ = 0.000 to 0.500, 0.600 to 0.900, and 0.925 to 1.000. On the behalf of each group, the mixtures at *w*_LLL_ = 0.417, 0.667, and 0.950 are selectively explained for the phase-transition behavior as follows.

In the mixture at *w*_LLL_ = 0.417, the transitional SOS α_2_-(2 + 3) crystals have already dissolved at the start of heating, so that only SOS γ-3 and LLL β′-2 crystals exist at the initial stage of heating. After the growth of the SOS γ-3 crystals without the fluctuating DSC heat flow, LLL β_2_-2 crystals occur at the partial expense of the LLL β′-2 crystals, giving one exothermic effect at the phase-transition temperature of 26.9 °C. With further heating, the SOS γ-3 crystals and the residual LLL β′-2 crystals with improved perfection melt and the LLL β_2_-2 crystals grow, resulting in one endothermic effect at the phase-transition temperature of 34.2 °C. The higher phase-transition temperatures, due to the melting of the resultant LLL β_2_-2 crystals, the conversion of the remaining LLL β_2_-2 crystals to LLL β_1_-2 crystals, and the melting of the LLL β_1_-2 crystals are not available: this is because the corresponding endothermic effect appears as a peak shoulder in the DSC thermogram, as indicated by a red arrow in the right part of Figure 1a.

In the mixture at *w*_LLL_ = 0.667, the phase-transition behavior starts from the solid phase consisting of transitional SOS α_2_-(2 + 3) crystals, SOS γ-3 crystals, and LLL β′-2 crystals. Compared to the mixture at *w*_LLL_ = 0.417, a larger number of phase-transition points are found in the phase behavior. A pair of endothermic and exothermic effects at phase-transition temperatures of 19.3 and 20.6 °C provide evidence that the transitional SOS α_2_-(2 + 3) crystals convert to SOS γ-3 crystals via melt-mediated transformation. Likewise, a pair of endothermic and exothermic effects at phase-transition temperatures of 29.7 and 31.0 °C provide evidence that the LLL β′-2 crystals partly convert to LLL β_2_-2 crystals via melt-mediated transformation. The endothermic effect at the phase-transition temperature of 33.9 °C corresponds to the melting of the SOS γ-3 crystals. The exothermic effect at the phase-transition temperature of 37.8 °C is due to the melt-mediated transformation from the residual LLL β′-2 crystals with improved perfection to LLL β_2_-2 crystals, where the exothermic heat of crystallization prevails over the endothermic heat of fusion. The endothermic effect at the phase-transition temperature of 43.6 °C includes a sequence of melting of the LLL β_2_-2 crystals, polymorphic transformation of the LLL crystals from β_2_-2 to β_1_-2, and the melting of the resultant LLL β_1_-2 crystals.

In the mixture at *w*_LLL_ = 0.950, LLL β′-2 crystals incorporating SOS as a solid solution solely exist at the initial stage of heating. The LLL β′-2 crystals partly convert to LLL β_2_-2 crystals probably via melt-mediated transformation, giving the exothermic effect at the phase-transition temperature of 32.1 °C. The other exothermic effect at the phase-transition temperature of 38.5 °C is due to the melt-mediated transformation from the residual LLL β′-2 crystals with improved perfection to LLL β_2_-2 crystals. The endothermic effect at the phase-transition temperature of 45.7 °C includes a sequence of melting of LLL β_2_-2 crystals, the polymorphic transformation of LLL crystals from β_2_-2 to β_1_-2, and the melting of resulting LLL β_1_-2 crystals.

#### 3.1.3. Heating Phase Behavior after Aging

Figure 10b indicates that the solid phase of the mixtures is stabilized by aging, leading to elevated phase-transition temperatures as well as the occurrence of SOS β_2_-3 and LLL β_2_-2 crystals at the initial stage of heating. The melt crystallization with constant-rate cooling was interrupted at 15 °C by subsequent aging including preliminary isothermal cooling at 15 °C for 30 min. Nevertheless, the aged mixtures exhibit a phase diagram similar to that of the non-aged mixtures. This indicates the possibility that melt-crystallization memory is inherited even after the long-term storage of the mixtures. Accordingly, the phase diagram of the aged mixtures is also divided into three areas of the mixtures at *w*_LLL_ = 0.000 to 0.500, 0.600 to 0.900, and 0.925 to 1.000, whose phase-transition behavior is explained by representative mixtures at *w*_LLL_ = 0.417, 0.667, and 0.950.

In the mixture at *w*_LLL_ = 0.417, the initially coexisting SOS β_2_-3 and LLL β_2_-2 crystals melt simultaneously, giving only one endothermic effect at the phase-transition temperature of 39.9 °C.

In the mixture at *w*_LLL_ = 0.667, the phase-transition behavior starts from the solid phase consisting mainly of SOS β_2_-3 and LLL β_2_-2 crystals. The endothermic effect at the phase-transition temperature of 33.3 °C indicates melting of the contaminated SOS γ-3 crystals, although no corresponding peaks are found in the SR-XRD data (Figure 6). This contradiction may result from the difference in detection sensitivity between DSC and SR-XRD. The endothermic effect at the phase-transition temperature of 35.8 °C is due to the polymorphic transformation of LLL crystals from β_2_-2 to β_1_-2, where the endothermic heat of fusion overcomes exothermic heat of crystallization; therefore, the manner of this transformation is attributable to melt mediation. The endothermic effect at the phase-transition temperature of 39.5 °C means the melting of the SOS β_2_-3 crystals. The endothermic effect at the phase-transition temperature of 43.5 °C corresponds to the melting of the LLL β_1_-2 crystals.

In the mixture at *w*_LLL_ = 0.950, SOS β_2_-3 and LLL β_2_-2 crystals coexist at the initial stage of heating. A pair of endothermic and exothermic effects at phase-transition temperatures of 37.2 and 38.5 °C means that the LLL β_2_-2 crystals convert to LLL β_1_-2 crystals via melt-mediated transformation. The endothermic effect at the phase-transition temperature of 45.5 °C is due to the melting of the LLL β_1_-2 crystals. Although the melting of the SOS β_2_-3 crystals is confirmed from ~37 to ~41 °C in the SR-XRD measurement (see Section 2.2.4), the endothermic effect is likely to be canceled by the exothermic effect, due to the occurrence of LLL β_1_-2 crystals; this also is true of the mixtures at *w*_LLL_ = 0.800 to 0.975.

### 3.2. Solubility Limit of SOS in SOS/LLL

In the metastable condition of cooling-and-heating, the solid-solubility limit of SOS in SOS/LLL lies between *w*_LLL_ = 0.900 and 0.925. In contrast, the solid-solubility limit moves to between *w*_LLL_ = 0.975 and 1.000 after aging at 25 °C for two weeks. The difference in solid-solubility limits means that aging narrows the solid-solution area by at least 5% in terms of the mass fraction in SOS/LLL. In other words, at least 5% SOS mass fraction, incorporated in LLL crystals as a solid solution, separates from the LLL crystals and crystallizes to form a new solid phase during aging.

Such phase separation and crystallization of the separated phase are considered the crucial cause of fat bloom in CBS-based compound chocolate. Large change in the solid-solubility limit combined with a mild crystallization condition for the separated phase may accelerate the formation of coarse crystals, resulting in fat bloom.

From these results, two ideas may be considered to prohibit fat bloom in CBS-based compound chocolate. The first one is to expand the solid-solution area after aging so that the solid-solution limit of CB remains far from the CBS-rich area. This may, however, hardly be achieved and the solid-solution area is narrowed by aging, as the present study shows. An alternative idea is to promote the polymorphic transformation of CBS and CB crystals to the most stable forms soon after the melt crystallization.

### 3.3. Future Research Directions

In this study, the binary phase behavior of SOS/LLL was examined under limited thermal conditions without other external factors. As main component TAGs, CB contains POS, SOS, and POP whereas CBS typically contains LLL, LLM, and LMM. To clarify the mechanisms of eutectic formation between CB and CBS and such associated adverse effects as fat bloom, further investigation of the mixing phase behavior of these TAGs in binary or multi-component systems is required. For practical applications, influences of external factors, including diverse thermal conditions and shear effect, on the mixing phase behavior should be considered as well. We believe that determining these mechanisms will help confectionery manufacturers develop new technologies that provide CB-based compound chocolate with fat-bloom resistance.

To retard fat-bloom formation in CBS-based compound chocolate, emulsifiers such as sorbitan tristearate have been used for a long time [28,51]. CBS with the TAG composition modified by means of fat blending and interesterification is promising for avoiding unnecessary items on food labels of chocolate [33,51,52]. Findings in this study will be especially helpful in developing fat-blending techniques for novel CBS products.

## 4. Materials and Methods

### 4.1. Materials and Sample Preparation

As TAG materials, SOS with 99% purity was purchased from Tsukishima Food Industry (Tokyo, Japan) and LLL with ≥ 99% purity was purchased from Sigma-Aldrich (St. Louis, MO, USA). These TAGs were used as received without further purification.

Mixtures of SOS/LLL were obtained by melt-mixing SOS with LLL at several mass fractions of LLL (*w*_LLL_): specifically, *w*_LLL_ = 0.000, 0.111, 0.200, 0.333, 0.417, 0.500, 0.600, 0.667, 0.800, 0.889, 0.900, 0.925, 0.950, 0.975, and 1.000. For example, the mixture at *w*_LLL_ = 0.667 contained SOS and LLL at a mass ratio of SOS/LLL = 0.333/0.667. The melt-mixing was conducted with vortex stirring in glass vials immediately after heating the vials above 80 °C.

### 4.2. Thermal Treatment for Measurements

For DSC and SR-XRD measurements, two types of samples were prepared from the SOS/LLL mixtures. Non-aged samples were available without any thermal pretreatment. Aged samples were given thermal pretreatment as follows: (i) heating at 80 °C for 10 min, (ii) cooling to 15 °C at a rate of 2 °C/min, (iii) isothermally cooling at 15 °C for 30 min, (iv) heating up to 25 °C at a rate of 5 °C/min, then (v) annealing at 25 °C for two weeks. Steps (i)–(iv) were completed using a thermal control device 10,021 (Linkam Scientific Instruments, Tadworth, UK). Step (v) was done in an incubator MIR-152 (Sanyo electric, Osaka, Japan).

During the measurements, the non-aged samples were heated at 80 °C for 10 min, cooled to 0 °C at a rate of 2 °C/min, then reheated to 80 °C at a rate of 5 °C/min; the aged samples were heated from 25 to 80 °C at a rate of 5 °C/min.

### 4.3. DSC Measurements

DSC measurements were conducted using heat-flux DSC Q100 (TA Instruments, New Castle, DE, USA). Temperature and heat flow were calibrated with reference to the melting points and enthalpies of indium and *n*-decane standards. Each SOS/LLL mixture (~3 mg) in the molten state was weighed on an aluminum pan and hermetically sealed with the pair lid, giving a sample pan. After being subjected to the aging process as necessary, the sample pan and an empty pan for reference were introduced to the thermally controlled measurement chamber where dry nitrogen gas flowed at a constant rate of 25 mL/min.

The measurement was repeated at least three times for each mixture. The thermograms obtained were analyzed for the peak-top temperatures using the software included in the DSC apparatus.

### 4.4. SR-XRD Measurements

SR-XRD measurements were carried out at a beamline BL-6A of Photon Factory, a synchrotron radiation facility of the High Energy Accelerator Research Organization (KEK) in Tsukuba, Japan. X-rays with a wavelength of 0.15 nm were applied to a sample cell, and the SAXS (WAXS) from the sample therein were simultaneously detected by PILATUS 1M (100K (Dectris, Baden-Daettwil, Switzerland)). The sample-to-detector distance was calibrated using a silver behenate standard.

The sample cell was made with a 1.5 mm-thick aluminum plate, having a 2 mm diameter round-hole to be filled with the SOS/LLL mixtures, and X-ray transmission windows of 25 μm thick polyimide films to seal both sides of the hole. After being aged as necessary, the sample cell was placed on the temperature-controlled stage of a thermal control device 10002L (Linkam Scientific Instruments, Tadworth, UK); in the measurement setup, the stage was vertically installed in front of the detectors. The SAXS and WAXS data were acquired every 6 s with 5 s X-ray exposure time and 1 s interval. Topographic plots of the SAXS and WAXS profiles were performed using OriginPro 2020b software (OriginLab, Northampton, MA, USA).

## 5. Conclusions

This study precisely analyzed the eutectic mixing behavior of SOS and LLL, as a typical model case of the mixture of CB and CBS. Using DSC and SR-XRD, the SOS/LLL mixtures were examined for crystallization kinetics, polymorphic phase-transition, and the solid-solubility limit of SOS. Integrating the acquired results, we obtained diagrams for the binary phase behavior of SOS/LLL that are similar to those of CB/CBS. The major findings on the mixing behavior of SOS/LLL are:

1. Under thermodynamically metastable conditions, SOS and LLL form eutectics in their metastable polymorphs, allowing the occurrence of a compatible solid solution;
2. The eutectic nature occurring in metastable conditions is retained even after the polymorphic stabilization of SOS and LLL crystals by aging;
3. The polymorphic stabilization narrows the solid-solution area of SOS in LLL crystals, indicating that SOS incorporated in LLL crystals as a solid solution separates from the LLL crystals and crystallizes to form a new solid phase.

Such phase separation and crystallization of the separated phase are considered the crucial cause of fat bloom in CBS-based compound chocolate. To solve this problem, we hope to develop retardation techniques against phase separation by applying new ways of fat blending: for example, molecular compound crystal formation [53].

## Figures and Tables

**Table 1:** Crystal polymorphs of 1,3-distearoyl-2-oleoyl-*sn*-glycerol (SOS) and trilaurin (LLL).

	Polymorph ^1^	Melting Point (°C)	Long Spacing (nm)	Short Spacing (nm)
SOS ^2^	sub α-2	—	5.00	0.42, 0.41
	α-2	23.5	4.83	0.421
	γ-3	35.4	7.05	0.472, 0.450, 0.388, 0.363
	β′-3	36.5	7.00	0.430, 0.415, 0.402, 0.395, 0.383, 0.370
	β_2_-3	41.0	6.50	0.458, 0.400, 0.390, 0.375, 0.367, 0.357
	β_1_-3	43.0	6.50	0.458, 0.402, 0.397, 0.385, 0.380, 0.365
LLL ^3^	α-2	15.0	3.5	0.42
	β′-2	35.0	3.2	0.42, 0.38
	β-2	46.5	3.1	0.46, 0.39, 0.38

^1^ Polymorphs are represented by sub-cell and chain-length structure (2: double and 3: triple). ^2^ Data are transferred from previous studies for sub α-2 [39] and other polymorphs [40]. ^3^ Data are transferred from the previous study [41].

## Supplementary Materials

The following are available online. Table S1: Peak-top temperatures (°C) of DSC exothermic and endothermic peaks in Figure 1, Figure S1: Topographic plots of SR-XRD data for SOS/LLL (*w*_LLL_ = 0.417), taken during cooling at a rate of 2 °C/min and subsequent heating at a rate of 5 °C/min: (a) SAXS; and (b) WAXS, Figure S2: Representative SR-XRD profiles of SOS/LLL (*w*_LLL_ = 0.417) in the heating process of Figure S1: (a) SAXS; and (b) WAXS. Unit: nm.
