# Supplementary material for: Binary Phase Behavior of 1,3-Distearoyl-2-oleoyl-sn-glycerol (SOS) and Trilaurin (LLL)
Source: Molecules. 2020 Nov 14;25(22):5313. doi: 10.3390/molecules25225313 (PMC7698300; doi:10.3390/molecules25225313)
Supplement: Supplementary file 1 [file molecules-25-05313-s001.pdf]

molecules

MDPI

Article

Binary Phase Behavior of 1,3-Distearoyl-2-oleoyl-*sn*-glycerol (SOS) and Trilaurin (LLL)

Shinichi Yoshikawa [1], Shimpei Watanabe [1], Yoshinori Yamamoto [2] and Fumitoshi Kaneko [2,*]

[1] Research Institute for Creating the Future, Fuji Oil Holdings Inc., Izumisano 598-8540, Japan; yoshikawa.shinichi@so.fujioil.co.jp (S.Y.); watanabe.shimpei@so.fujioil.co.jp (S.W.)

[2] Graduate School of Science, Osaka University, Toyonaka 560-0043, Japan; yamamotoy16@chem.sci.osaka-u.ac.jp

* Correspondence: toshi@chem.sci.osaka-u.ac.jp; Tel.: +81-6-6850-5453

Academic Editor: Sato Kiyotaka

Received: 29 October 2020; Accepted: 12 November 2020; Published: date

Table S1. Peak-top temperatures [1,2] (°C) of DSC exothermic and endothermic peaks in Figure 1.

	Without aging treatment (Figure 1a)									After aging treatment (Figure 1b)					
w_{LLL}	Cooling		Heating							Heating					
**0.000**	22.6			27.9			36.2							41.8	
0.111	22.0	17.9				29.3	35.3						39.1	41.1	
0.200	20.9	18.5				29.3	34.6							39.5	
0.333	20.0	18.5				28.6	34.3							39.6	
0.417	18.5	17.6				26.9	34.2							39.9	
0.500	17.5	17.0				26.4	33.9		41.4					39.7	
0.600		11.6	19.6	20.7	29.8	31.1	33.9		42.8	33.1	35.7			39.6	42.7
0.667		12.6	19.3	20.6	29.7	31.0	33.9	37.8	43.6	33.3	35.8			39.5	43.5
0.800		12.8		21.1	29.3	31.9	33.9	37.6	44.5	33.5	35.6				43.5
0.889		14.9				30.0	33.5	37.8	45.1	32.8	36.0				44.3
0.900		14.8				30.3	33.4	37.9	45.3	32.9	36.3				45.3
0.925		13.5				29.5		37.9	45.4		36.8	38.2			45.4
0.950		14.7				32.1		38.5	45.7		37.2	38.5			45.5
0.975		16.4				31.7		39.1	46.0			39.6			45.9
1.000		18.0				27.1			45.8						46.1

[1] Standard deviations between peak-top temperatures in the multiple measurements are 1.2 °C or less.

[2] Peak-top temperatures in blue or red color denote exothermic or endothermic peaks, respectively.

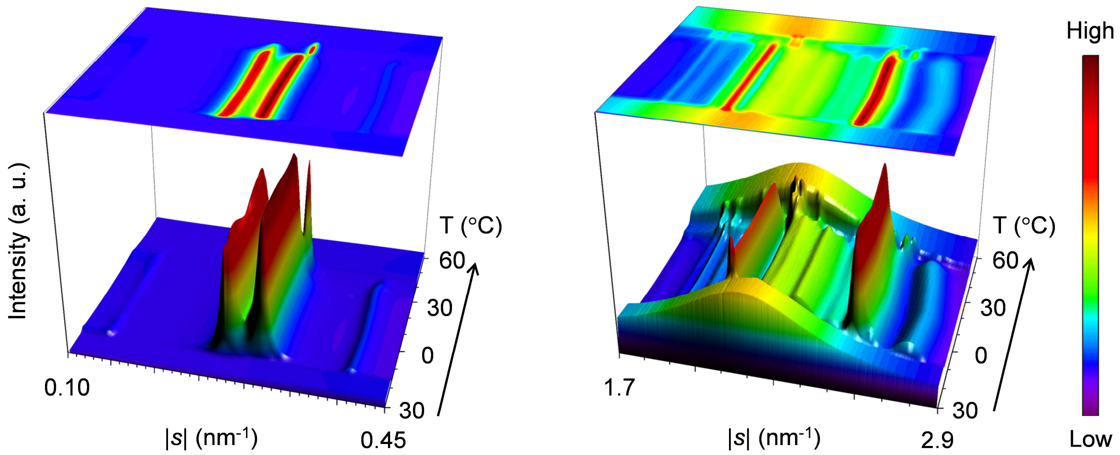

(**a**) (**b**)

Figure S1. Topographic plots of SR-XRD data for SOS/LLL (w_{LLL} = 0.417), taken during cooling at a rate of 2 °C/min and subsequent heating at a rate of 5 °C/min: (a) SAXS; and (b) WAXS.

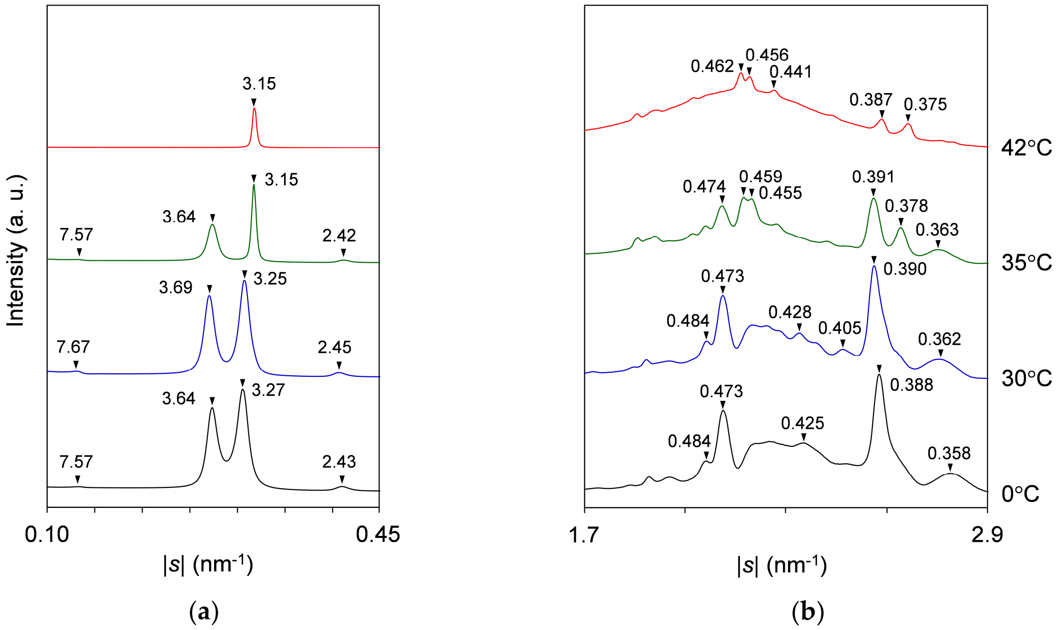

Figure S2. Representative SR-XRD profiles of SOS/LLL (w_{LLL} = 0.417) in the heating process of Figure S1: (a) SAXS; and (b) WAXS. Unit: nm.

Sample Availability: Samples of the compounds are not available from the authors.

Publisher's Note: MDPI stays neutral with regard to jurisdictional claims in published maps and institutional affiliations.

© 2020 by the authors. Submitted for possible open access publication under the terms and conditions of the Creative Commons Attribution (CC BY) license (http://creativecommons.org/licenses/by/4.0/).
